# Prediction of hepatocellular carcinoma response to radiation segmentectomy using an MRI-based machine learning approach

**DOI:** 10.1007/s00261-024-04606-z

**Published:** 2024-10-26

**Authors:** Daniel Stocker, Stefanie Hectors, Brett Marinelli, Guillermo Carbonell, Octavia Bane, Miriam Hulkower, Paul Kennedy, Weiping Ma, Sara Lewis, Edward Kim, Pei Wang, Bachir Taouli

**Affiliations:** 1 BioMedical Engineering and Imaging Institute, Icahn School of Medicine at Mount Sinai, New York, NY USA; 2 Institute of Diagnostic and Interventional Radiology, University Hospital Zurich, University of Zurich, Rämistrasse 100, 8091 Zurich, Switzerland; 3 Department of Diagnostic, Molecular and Interventional Radiology, Icahn School of Medicine at Mount Sinai, New York, NY USA; 4 Division of Interventional Radiology, Department of Radiology, Memorial Sloan Kettering Cancer Center, New York, NY USA; 5 Department of Radiology, University Hospital Virgen de la Arrixaca, Murcia, Spain; 6 Department of Genetics and Genomics Sciences, Icahn School of Medicine at Mount Sinai, New York, NY USA; 7 Liver Cancer Program, Tisch Cancer Institute, Icahn School of Medicine at Mount Sinai, New York, NY USA

**Keywords:** Carcinoma, Hepatocellular, Magnetic resonance imaging, Yttrium radioisotopes

## Abstract

**Purpose:**

To evaluate the value of pre-treatment MRI-based radiomics in patients with hepatocellular carcinoma (HCC) for the prediction of response to Yttrium 90 radiation segmentectomy.

**Methods:**

This retrospective study included 154 patients (38 female; mean age 66.8 years) who underwent contrast-enhanced MRI prior to radiation segmentectomy. Radiomics features were manually extracted on volumes of interest on post-contrast T1-weighted images at the portal venous phase (PVP). Tumor-based response assessment was evaluated 6 months post-treatment using mRECIST. A logistic regression model was used to predict binary response outcome [complete response at 6 months with no-re-treatment (response group) against the rest (non-response group, including partial response, progressive disease, stable disease and complete response after re-treatment within 6 months after radiation segmentectomy) using baseline clinical parameters and radiomics features. We accessed the value of different sets of predictors using cross-validation technique. AUCs were compared using DeLong tests.

**Results:**

A total 168 HCCs (mean size 2.9 ± 1.7 cm) were analyzed in 154 patients. The response group consisted of 113 HCCs and the non-response group of 55 HCCs. Baseline clinical parameters (AUC 0.531; sensitivity, 0.781; specificity, 0.279; positive predictive value (PPV), 0.345; negative predictive value (NPV), 0.724) and AFP (AUC 0.632; sensitivity, 0.833; specificity, 0.466; PPV, 0.432; NPV, 0.851) showed poor performance for response prediction. The model using a combination of radiomics features and clinical parameters/AFP showed the best performance (AUC 0.736; sensitivity, 0.706; specificity, 0.662; PPV 0.504; NPV, 0.822), significantly better than the clinical model (p < 0.001) or AFP alone (p < 0.001).

**Conclusion:**

The combination of radiomics features from pre-treatment MRI with clinical parameters and AFP showed fair performance for predicting HCC response to radiation segmentectomy, better than that of AFP. These results need further validation.

**Supplementary Information:**

The online version contains supplementary material available at 10.1007/s00261-024-04606-z.

## Introduction

Liver cancer is the sixth most commonly diagnosed cancer and the third leading cause of cancer death worldwide, predominantly affecting males [1]. Accounting for 75–85% of cases, hepatocellular carcinoma (HCC) is the most common primary malignant liver cancer with variable incidence rates depending on the geographical location [1]. During the multistep hepatocarcinogenesis a typical shift from a portal venous dominated vascular supply to an arterial dominated vascular supply of the lesion is usually observed. The higher percentage of arterial supply in HCC compared to liver parenchyma makes HCC a good target for transarterial directed locoregional therapies such as transarterial chemoembolization (TACE) or Yttrium 90 (^90^Y) radioembolization (TARE, also called selective internal radiation therapy or SIRT). Radiation segmentectomy, a special type of locoregional therapy with targeted application of ^90^Y is typically administered to a maximum of two specific hepatic segments. Both TACE and TARE have established roles in patients with intermediate HCC, in patients who are ineligible for liver transplantation or as neoadjuvant treatment to bridge the time to transplantation [2–4]. So far, TACE and TARE show similar overall survival rates, with longer time to progression, better tumor control and higher quality of life with TARE [5–7].

Reduced or lack of enhancement of the treated lesion is an important imaging feature of treatment response in patients with HCC who undergo liver directed therapy. However, this feature is only seen late after TARE (compared to TACE), therefore additional imaging features are needed to assess early response.

Radiomics is a high-throughput quantitative image analysis method to extract a large number of features from medical images using different data characterization algorithms, also including histogram and texture analysis algorithms [8]. Texture analysis is an objective method which allows for evaluation of gray-level image features in a specific region or volume of interest (VOI) that cannot be assessed by the naked human eye [9, 10]. Texture analysis uses different mathematical models, which, among others, take grey-level intensity, pixel position and distances between pixels into account to, for example, assess tumor heterogeneity. So far, studies have investigated the use of magnetic resonance imaging (MRI) texture analysis to characterize HCC [11–13], predict HCC recurrence [14, 15] and progression [16] and to chatacterize focal liver lesions [17, 18].

As of now, a few studies looked at risk prediction models for patients with HCC treated with TARE/radiation segmentectomy [19–24]. Studies investigating quantitative baseline MRI features to predict response of HCC to TARE are needed to optimize patient selection and to assess the need of additional (systemic) therapy.

The purpose of our study was to evaluate the value of pre-treatment clinical parameters and quantitative MRI features in HCC tumors for the prediction of tumor response to radiation segmentectomy.

Results from 73 and 78 patients from the current cohort were reported in previous studies [21, 25]. Although there are some similarities regarding objectives in a previous study [21], the current study is different as it did not incorporate post-treatment MRI data, the radiomics extraction involved different MRI sequences and different segmentations approaches and analysis.

## Materials and methods

This HIPAA compliant, single center retrospective study was approved by the institutional review board which waived the requirement for written informed consent.

### Study population

**Fig. 1:**
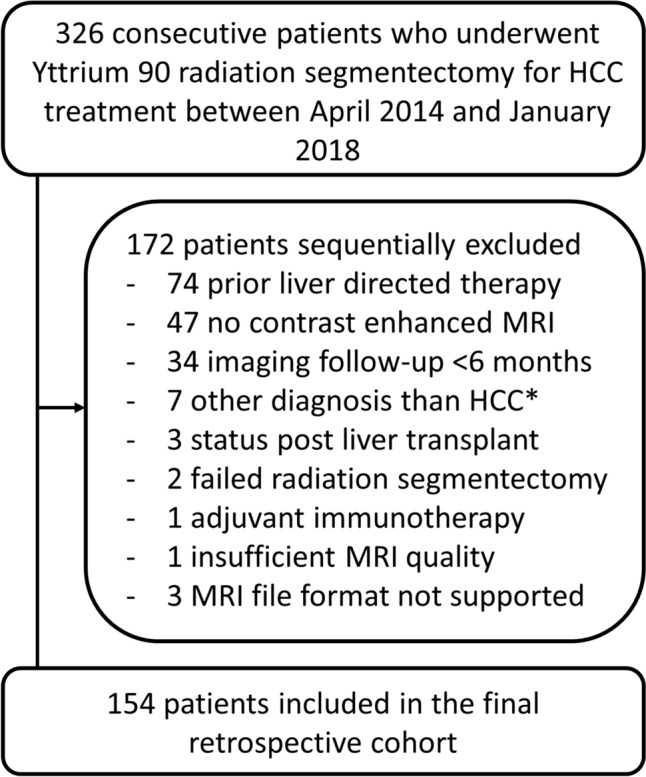
Population flowchart. *Based on pathology after radiation segmentectomy. *HCC* hepatocellular carcinoma, *MRI* magnetic resonance imaging

We enrolled 326 consecutive patients who underwent Yttrium 90 radiation segmentectomy for HCC treatment between April 2014 and January 2018 from the interventional radiology database. The diagnosis of HCC was based on imaging (Liver Imaging Reporting and Data System, LI-RADS) and multidisciplinary tumor board decision. A clinical chart is shown in Fig. 1 Demographic, clinical and laboratory parameters for all included patients were collected from their electronic medical record.

The final study population consisted of 154 patients (38 female/116 male; mean age 66.8 ± 9.7 years). Patient demographics and clinical parameters are summarized in Table 1. The mean interval between baseline MRI and radiation segmentectomy was 52.8 ± 18.0 days (range 7–115 days).

**Table 1:** Patient demographics and clinical parameters (154 patients with 168 HCC lesions)

Gender: Female/Male (n)	38 (24.7%) / 116 (75.3%)
Age (mean ± SD,)	66.8 ± 9.7 years
*Race/ethnicity (n)*	
White non-Hispanic	62 (40.3%)
White Hispanic	31 (20.1%)
Black	24 (15.6%)
Asian	24 (15.6%)
Other	13 (8.4%)
*Etiology of liver disease (n)*	
HCV	71 (46.1%)
MASH	23 (14.9%)
HBV	23 (14.9%)
ETOH	19 (12.3%)
Cryptogenic cirrhosis	7 (4.5%)
Autoimmune hepatitis	3 (1.9%)
Other	4 (2.6%)
No underlying liver disease	4 (2.6%)
*Child–Pugh class (n)*	
A	124 (80.5%)
B	29 (18.9%)
C	1 (0.6%)
MELD score (mean ± SD)	9.9 ± 3.6
*BCLC stage (n)*	
0	11 (7.1%)
A	106 (68.8%)
B	27 (17.5%)
C	9 (5.8%)
D	1 (0.6%)
AFP at baseline (median, range)	9.4 (4.4–66.2) ng/ml
Lesion size (mean ± SD)	2.9 ± 1.7 cm
*Lesion location (n)*	
Right lobe/left lobe	124 (73.8%)/44 (26.2%)
Patients with multiple lesions (n)	13 (8.4%)^a^
Number of lesions/patient (mean ± SD)	1.1 ± 0.3
Patients with macrovascular invasion (n)	6
Y90 dose (mean ± SD)	1.74 ± 0.87 GBq

Nominal variables are presented as frequencies with percentages in parentheses and numerical variables as mean ± standard deviation or median with interquartile range in parentheses

*HCV* hepatitis C virus, *HBV* hepatitis B virus, *MASH* metabolic associated steatohepatitis, *ETOH* alcohol-related liver disease, *MELD* model for end-stage liver disease, *BCLC* Barcelona clinic liver cancer, *AFP* Alpha Fetoprotein, *HCC* hepatocellular carcinoma

^a^Eleven patients had 2 lesions and 2 patients had 3 lesions

### MRI technique

Baseline MRI was performed on 1.5 T (n = 108) or 3 T systems (n = 46) from several vendors using a hepatobiliary [n = 129 (gadoxetic acid, n = 128 and gadobenic acid, n = 1)] or an extracellular contrast agent (n = 25; gadobutrol, n = 10; gadoversetamide, n = 5; gadoteridol, n = 5; gadopentetic acid, n = 3; gadoteric acid, n = 1; gadodiamide, n = 1). Contrast-enhanced T1-weighted (T1W) images in the arterial and portal venous phase (PVP), T2-weighted images as well as in- and opposed phase images were available for all patients, while diffusion-weighted images (DWI) and hepatobiliary phase (HBP) images were not available for 13 and 25 patients, respectively. The acquired b-values ranged from 0 to 1000 s/mm^2^ with a minimal high b-value of at least 500 s/mm^2^. More detailed information regarding MRI parameters are found in the Supplementary Material.

### Yttrium-90 radiation segmentectomy

Radiation segmentectomy was defined as transarterial ^90^Y infusion limited to two or less hepatic segments in one treatment session [26]. Procedure and dosage methodology specifics have been previously described [26–28]. In brief, to identify tumor feeding vessels a planning angiogram was performed prior to radiation segmentectomy. In the same session, 99Technetium‐macroaggregated albumin was injected for shunt detection and quantification. All patients were treated with 20–30 μm‐sized glass-based microspheres (TheraSphere, Boston Scientific International) to transarterially deliver ^90^Y to the target area. The injected activity (GBq) was dependent on tumor size and the lung shunt fraction with an intended target lesion dosing > 190 Gy. The mean injected activity during radiation segmentectomy was 1.74 ± 0.87 GBq (range 0.48–5.20 GBq).

### Parameters for demographic/clinical model

The following demographic and clinical parameters were used to develop a model: age (years), gender (male/female), race/ethnicity (White non-Hispanic, Black, Asian, White Hispanic, other), history of prior HCC (yes/no), serum AFP (ng/ml), MELD score, Child–Pugh score, number of target lesions, Yttrium 90 dose (GBq), underlying liver disease and Barcelona Clinic Liver Cancer stage.

### Tumor segmentation and radiomics extraction

One radiologist (reader 1, DS, a radiologist with 2 years post-training experience in abdominal imaging) placed three-dimensional free-hand volumes of interests (VOIs) covering the entire liver tumor on PVP T1W images, HBP images and apparent diffusion coefficient (ADC) maps from baseline MRI studies using freeware compatible with the Image Biomarker Standardization Initiative (IBSI) standard [29] (LIFEx v.5.1, www.lifexsoft.org) [30]. Image sequence selection was based on robustness of the sequence (PVP and HBP being less sensitive to motion artifacts compared to the arterial phase) and expected predictive value [31]. A second radiologist (reader 2, MH, an abdominal MRI fellow with 1 year of post-training experience) placed VOIs in the same fashion in a randomly chosen subset of 67 lesions. Reader 1 repeated the VOI placement more than 3 months after the initial readout in a randomly chosen subset of 50 lesions. We calculated the intraclass correlation coefficient (ICC) to assess inter- and intra-reader agreement for the extracted radiomics values. An ICC of 0.75 to 1 indicated excellent, 0.60 to 0.74 good, 0.40 to 0.59 fair, and less than 0.4 poor agreement [32]. Before VOI placement and feature extraction, image pre-processing was performed (Supplementary Material).

A total of 45 features from VOIs were extracted, consisting of conventional indices (n = 4), first-order features (also known as histogram features, n = 6), shape features (n = 3) and second-order features (gray-level co-occurrence matrix, GLCM, n = 7; neighborhood gray-level different matrix, NGLDM, n = 3; gray-level run length matrix, GLRLM, n = 11; gray-level zone length matrix, GLZLM, n = 11) (Supplementary Table 1).

### Feature selection and radiomic model building

Heavy tailed distribution was observed for many MRI radiomics features, which is not compatible to the requirement of regression model with Gaussian assumption. We performed a Shapiro-Wilks test to check for normality of all radiomics features and took log^2^ transformation of those features with extreme test p-value (< 10e^−18^). In order to model the relationship between response and predictors, radiomics feature selection was obtained by multivariate logistic regression with elastic net regularization [33] to deal with multiple cross related covariates and reduce the risk of overfitting of the data. The elastic net regularization is a combination of ridge and least absolute shrinkage and selection operator regression methods and automatically performs variable selection and continuous shrinkage. This method helps to select groups of correlated variables allowing to identify the best predictors when a set of predictors is much larger than the number of cases. Regularization parameters tuning and coefficients fitting in the regression model were determined by the default setting in the package [33].

### Endpoint: response assessment

Lesion response was assessed using mRECIST (modified Response Evaluation Criteria In Solid Tumors, Supplementary Material) [34] by an experienced radiologist (BT, with 17 years of post-training experience in abdominal imaging). While mRECIST primarily aims to determine overall disease status per patient [34], this study adopted the mRECIST principle for a per-lesion basis interpretation of tumor response as shown before [35, 36]. All post-radiation segmentectomy follow-up cross-sectional imaging studies up to 6 months were evaluated. Results from response assessment at 6 months were used for further analysis.

For the prediction model development, lesions were divided into two groups based on the response evaluation and the treatment plan changes: a response group (n = 113), including lesions with complete response (CR) without a second treatment on the same lesion within 6 months; a non-response group (n = 55), including all partial response (PR), stable disease (SD), progressive disease (PD) lesions, and lesions with CR but which underwent a second treatment within 6 months of the initial treatment.

### Statistical analysis

**Fig. 2:**
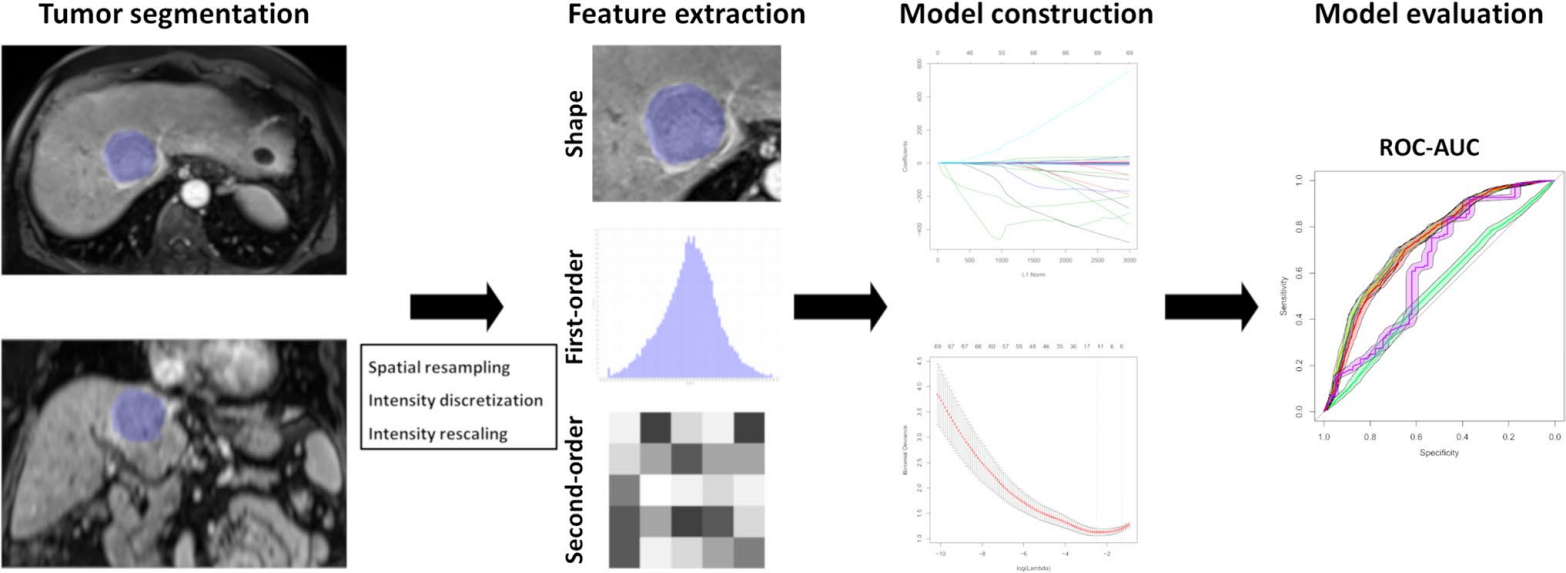
Overview of the workflow for model development

Prediction accuracy of the univariate regression models was evaluated by calculating ROC curve and corresponding AUC values using the response group and feature values as prediction. Prediction accuracy of the multivariate regression model was assessed by random cross validation with a 100 times re-sampling procedure. 20% of samples from each response group were sampled as prediction set and were predicted by the fitted model on the 80% remaining training samples. We combined the response and prediction in each re-sampling step to construct complete cross validation prediction results. ROC curve and corresponding AUC as well as sensitivity and specificity of the optimal cutoff value were calculated based on the complete cross validation prediction. AUCs were compared pairwise using De Long tests. The statistical analyses were performed using R statistical software (R version 3.5.1). Two-sided p-values < 0.05 were considered significant. The glmnet package in R was used in this study [33]. An overview of the workflow for the model development is presented in Fig. 2.

## Results

A total of 168 HCCs were evaluated in 154 patients. At 6 months follow-up, 127 lesions (75.6%) showed CR, 23 lesions (13.7%) PR, 7 lesions (4.2%) SD and 11 lesions (6.5%) PD. Fourteen lesions (8.3%) categorized as CR at 6 months underwent re-treatment with TARE before 6 months and were allocated in the non-response group. Therefore, the response group consisted of 113 lesions, including lesions with CR without re-treatment of the same lesion within 6 months; and the non-response group of 55 lesions, including lesions with PR, SD, PD and lesions with CR which underwent re-treatment within 6 months of the initial treatment.

### Inter- and intra-observer agreement

The inter- and intra-observer agreement was excellent for all radiomics features (ICC, 0.811–1) except for the inter-observer agreement of GLZLM_LGZE (ICC, 0.558) and inter- and intra-observer agreement of GLZLM_SZLGE (ICC, 0.441 and 0.692, respectively) (Supplementary Table 1).

### Prediction models

#### Univariate analysis

Univariate prediction accuracy of each single feature in all predictor sets (demographic/clinical features; MRI radiomics features) were calculated separately. Thirty-four of 71 features achieved statistical significance (p < 0.05).

MRI radiomics features from PVP had the highest prediction accuracy (mean AUC 0.627 ± 0.079) compared to demographic/clinical features (mean AUC 0.532 ± 0.032). The radiomics feature “SHAPE_Volume” demonstrated the highest AUC (0.745), indicating that lesions with lower tumor volumes are more likely to achieve CR. Among demographic/clinical variables, AFP showed the highest AUC (0.619), indicating that patients with lower AFP are more likely to achieve CR. Lesions in patients with BCLC stage A had more frequent CR while, in contrast, lesions in patients with BCLC stage B, C and D showed more frequently non-CR. Results from the univariate analysis are summarized in Supplementary Table 2.

#### Multivariate analysis

Different types of predictor sets were considered in the multivariate models: demographic/clinical features alone including AFP (Model A); AFP alone (Model B); radiomics from PVP alone (Model C); a combination of demographic/clinical features including AFP and radiomics from PVP MRI (Model D); a combination of AFP and radiomics from PVP MRI (Model E). Since we observed non-negligible missing values in HBP and ADC radiomics features, we were not able to include those in the regression model.

**Fig. 3:**
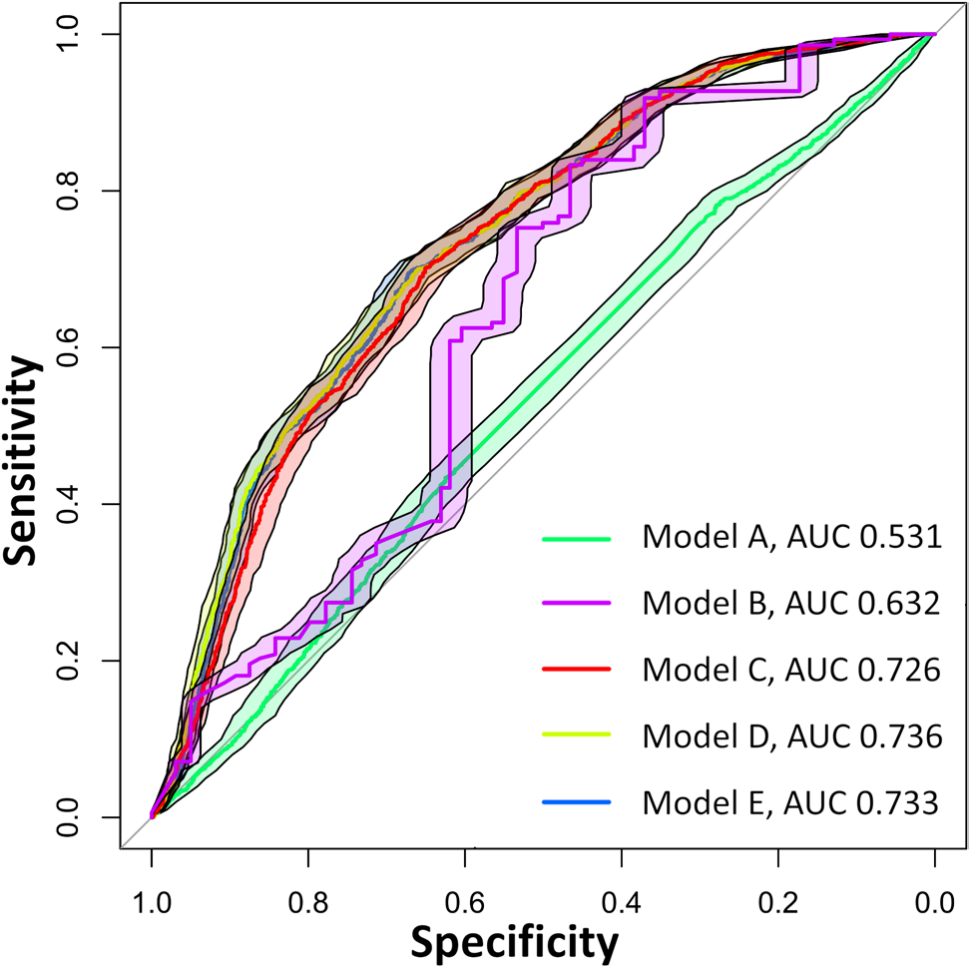
ROC for prediction of response. Model A, clinical and demographic features. Model B, AFP alone. Model C, radiomics from PVP MRI. Model D, clinical and demographic features + radiomics from PVP MRI. Model E, AFP alone + radiomics from PVP MRI. *AFP* Alpha Fetoprotein, *PVP* portal venous phase, *MRI* magnetic resonance imaging

The prediction Model C (radiomics from PVP alone) showed a significantly higher AUC (0.726) compared to Model A (demographic/clinical features, AUC 0.531; p < 0.001) and Model B (AFP alone, AUC 0.632; p < 0.001). Model D (combination of demographic/clinical features and radiomics from PVP, AUC 0.736) and Model E (combination of AFP and radiomics from PVP, AUC 0.733) showed a slightly, but not significantly improved performance compared to Model C (p = 0.358 and 0.558, respectively). The results from the multivariate analysis are summarized in Table 2 and Fig. 3.

**Table 2:** Performance and comparison of the performance of the different prediction models for HCC response using the De Long test

Performance	AUC	Sensitivity	Specificity	PPV	NPV	Youden Index
Model A	0.531	0.781	0.279	0.345	0.724	0.061
Model B	0.632	0.833	0.466	0.432	0.851	0.299
Model C	0.726	0.701	0.651	0.494	0.817	0.352
Model D	0.736	0.706	0.662	0.504	0.822	0.368
Model E	0.733	0.695	0.672	0.508	0.819	0.367

Comparison	Model A	Model B	Model C	Model D	Model E
Model A	–	< 0.001	< 0.001	< 0.001	< 0.001
Model B	< 0.001	–	< 0.001	< 0.001	< 0.001
Model C	< 0.001	< 0.001	–	0.558	0.358
Model D	< 0.001	< 0.001	0.358	–	0.098
Model E	< 0.001	< 0.001	0.558	0.098	–

Model A, clinical and demographic features

Model B, AFP alone

Model C, radiomics from PVP MRI

Model D, clinical and demographic features + radiomics from PVP MRI

Model E, AFP + radiomics from PVP MRI

*AFP* Alpha Fetoprotein, *PVP* portal venous phase, *MRI* magntic resonance imaging

**Fig. 4:**
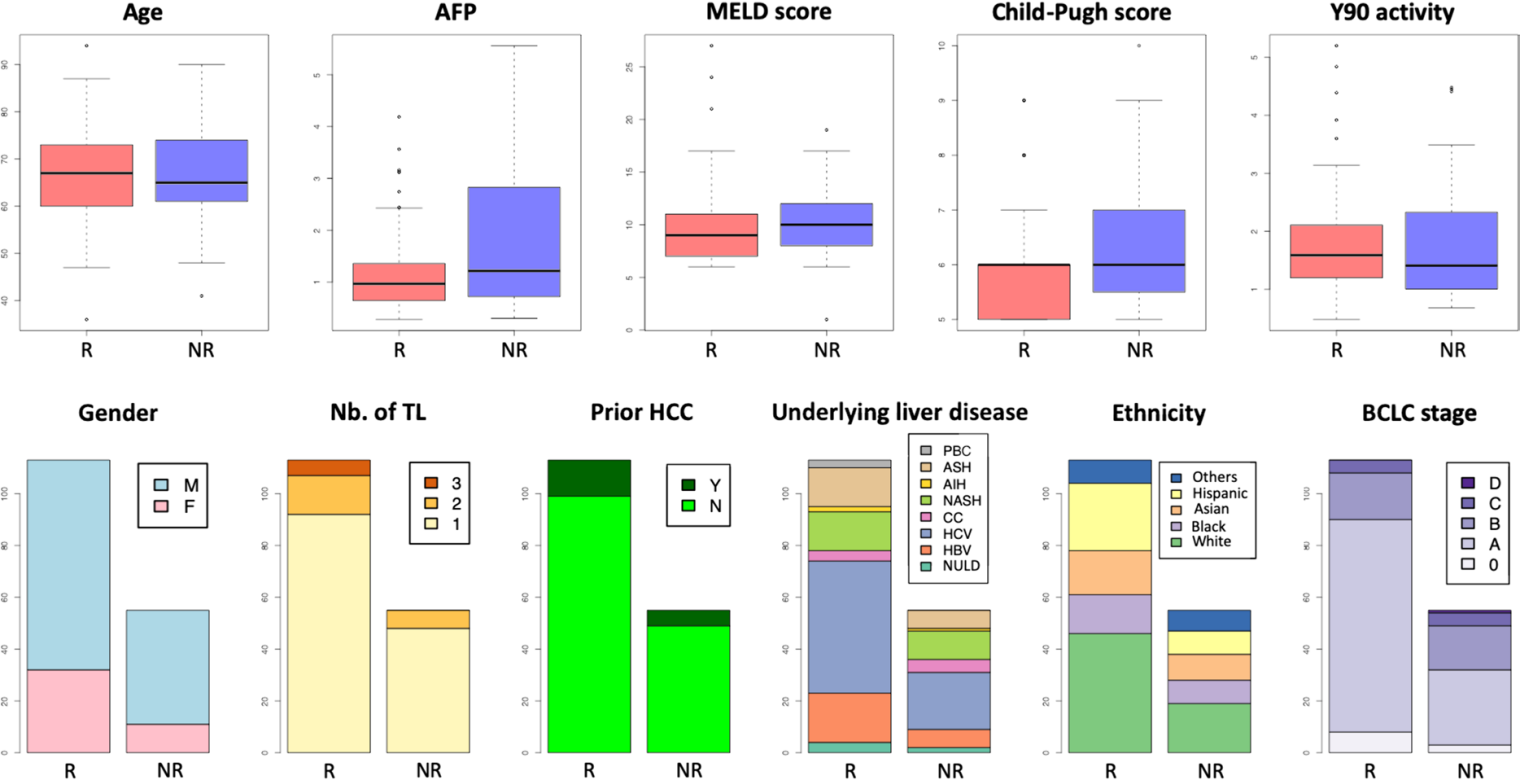
Box-plots and bar charts showing the distribution of demographic and clinical features for the response [(R, includes complete response (CR)] and non-response group [NR, includes partial response (PR), stable disease (SD), progressive disease (PD) lesions, and lesions with CR which underwent a second treatment within 6 months of the initial treatment]. Most demographic and clinical features show similar distribution between the response and non-response group. Statistically significant differences in the univariant analysis between the groups were only observed for AFP (p = 0.012), Child–Pugh score (p = 0.039), BCLC stage A (p = 0.011) and BCLC stage B (p = 0.025) (see also Supplementary Table 2). *AFP* Alpha Fetoprotein; *MELD* Model for End-Stage Liver Disease, *Y90* Yttrium-90, *Nb. of TL* Number of Target Lesions, *HCC* Hepatocellular Carcinoma, *BCLC stage* Barcelona Clinical Liver Cancer stage, *M* Male, *F* Female, *Y* yes, *N* no, *PBC* Primary Biliary Cirrhosis, *ASH* Alcohol Steatohepatitis, *NASH* Non-Alcoholic Steatohepatitis, *CC* Cryptogenic Cirrhosis, *AIH* Autoimmune Hepatitis, *HCV* Hepatitis C Virus, *HBV* Hepatitis B Virus, *NULD* No Underlying Liver Disease

**Fig. 5:**
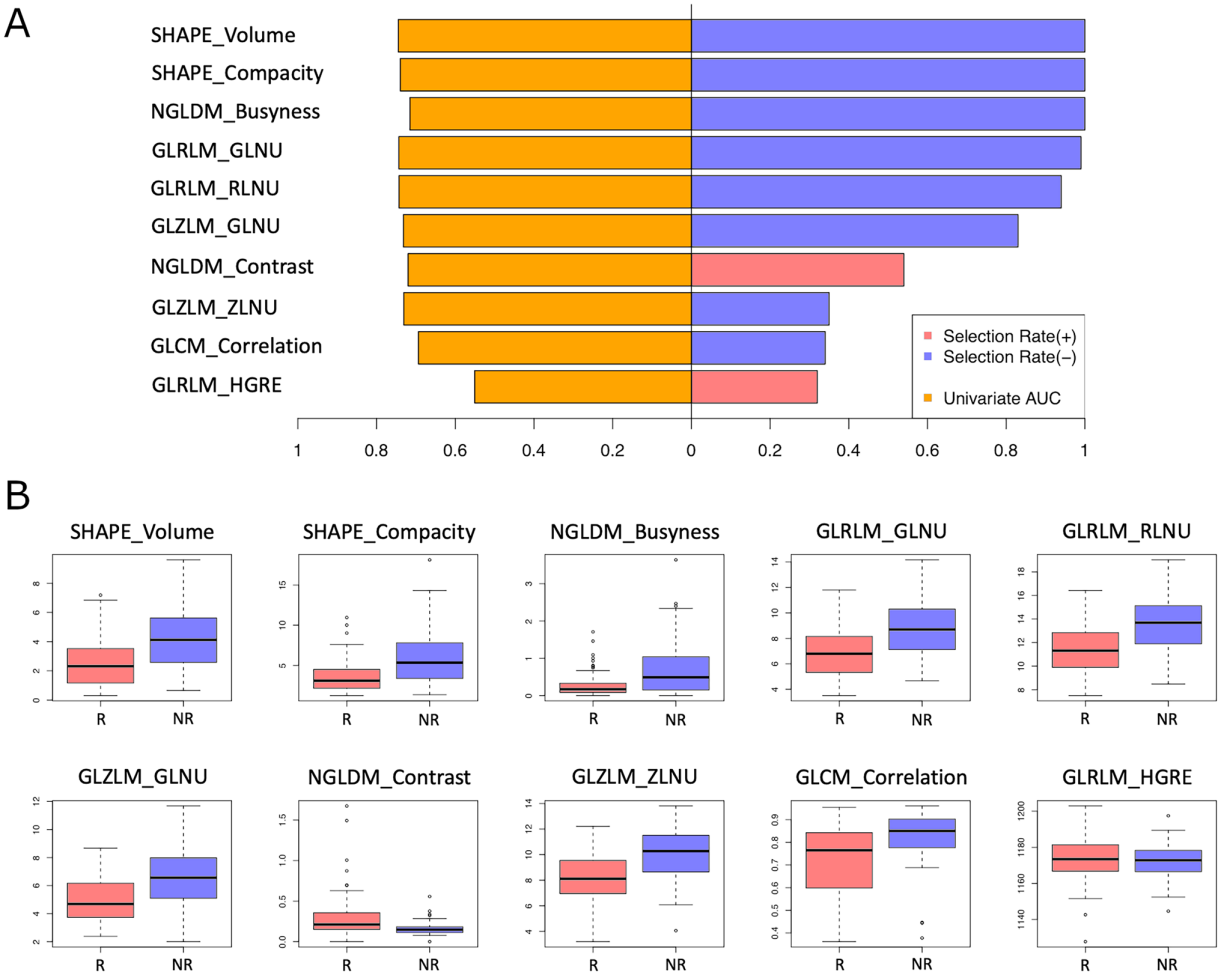
Model selection results based on random cross validation with a 100 times re-sampling procedure. **A** Selection rate as well as the univariate AUC of the top 10 most frequently selected radiomics features. **B** Distribution of the top 10 most frequently selected radiomics features for the response group [(R, includes complete response (CR)] and non-response group [NR, includes partial response (PR), stable disease (SD), progressive disease (PD) lesions, and lesions with CR which underwent a second treatment within 6 months of the initial treatment]

Among all the random sample cross validation iterations, we summarized the frequency with which each variable has been selected by the fitted model on training data in Table 3 and illustrated it in Figs. 4 and 5. The most commonly selected radiomics features from PVP MRI showed relatively higher accuracy in univariate prediction with AUCs > 0.7.

**Table 3:** Frequency of feature selection by the fitted model on training data for Model D (combination of clinical/demographic features and radiomics)

	Features	AUC	Selection frequency—total	Selection frequency—CR	Selection frequency—non-CR	Inter-reader agreement (ICC) (95%CI)	Intra-reader agreement (ICC) (95%CI)
Radiomics	SHAPE_Volume	0.745	1	0	1	1 (1–1)	1 (0.999–1)
	SHAPE_Compacity	0.740	1	0	1	0.999 (0.999–1)	0.999 (0.999–1)
	GLRLM_GLNU	0.744	1	0	1	1 (1–1)	1 (0.999–1)
	NGLDM_Busyness	0.715	1	0	1	1 (1–1)	0.999 (0.999–1)
	GLRLM_RLNU	0.743	0.99	0	0.99	1 (1–1)	1 (0.999–1)
	GLZLM_GLNU	0.732	0.74	0	0.74	1 (1–1)	1 (0.999–1)
	GLZLM_ZLNU	0.731	0.29	0	0.29	0.999 (0.999–1)	1 (0.999–1)
	NGLDM_Contrast	0.720	0.25	0.25	0	0.811 (0.693–0.884)	0.835 (0.709–0.906)
	GLCM_Correlation	0.694	0.18	0	0.18	0.951 (0.920–0.970)	0.966 (0.940–0.981)
	GLCM_Contrast	0.687	0.15	0.15	0	0.912 (0.857–0.946)	0.954 (0.919–0.974)
	GLRLM_HGRE	0.551	0.14	0.14	0	0.898 (0.834–0.937)	0.961 (0.932–0.978)
	GLRLM_SRHGE	0.650	0.14	0.14	0	0.934 (0.893–0.960)	0.966 (0.941–0.981)
	HISTO_Kurtosis	0.591	0.07	0.07	0	0.874 (0.794–0.922)	0.947 (0.907–0.970)
	HISTO_ExcessKurtosis	0.591	0.07	0.07	0	0.874 (0.794–0.922)	0.947 (0.907–0.970)
	GLZLM_SZE	0.621	0.07	0.07	0	0.936 (0.897–0.961)	0.926 (0.869–0.958)
	GLRLM_LGRE	0.595	0.05	0.05	0	0.878 (0.802–925)	0.924 (0.867–0.957)
	GLZLM_LZHGE	0.697	0.05	0	0.05	0.999 (0.999–1)	0.998 (0.997–0.999)
	GLZLM_ZP	0.659	0.04	0.04	0	0.951 (0.920–0.970)	0.974 (0.954–0.985)
	HISTO_Skewness	0.639	0.03	0	0.03	0.938 (0.899–0.962)	0.950 (0.912–0.972)
	GLCM_Dissimilarity	0.673	0.03	0.03	0	0.935 (0.894–0.960)	0.959 (0.928–0.977)
	GLRLM_SRLGE	0.599	0.03	0.03	0	0.878 (0.802–925)	0.920 (0.859–0.955)
	GLZLM_SZHGE	0.551	0.03	0.03	0	0.948 (0.916–0.968)	0.952 (0.916–0.973)
	CONVENTIONAL_mean	0.500	0.02	0.02	0	1 (1–1)	1 (1–1)
	GLZLM_LZE	0.691	0.02	0	0.02	0.999 (0.999–0.999)	0.998 (0.996–0.999)
	CONVENTIONAL_max	0.478	0.01	0.01	0	1 (1–1)	1 (1–1)
	NGLDM_Coarseness	0.739	0.01	0.01	0	0.959 (0.934–0.975)	0.952 (0.915–0.973)
	GLZLM_SZLGE	0.537	0.01	0	0.01	0.441 (0.091–0.656)	0.692 (0.457–0.825)
Clinical/demographic features	BCLC stage A	0.599	0.31	0.31	0	NA	NA
	BCLC stage D	0.509	0.22	0	0.22	NA	NA
	Child–Pugh score	0.591	0.14	0	0.14	NA	NA
	^90^Y dose	0.531	0.14	0.14	0	NA	NA
	AFP	0.619	0.11	0	0.11	NA	NA
	Ethnicity “other”	0.533	0.06	0	0.06	NA	NA
	Underlying liver disease “cryptogenic cirrhosis”	0.519	0.03	0	0.03	NA	NA
	Male gender	0.542	0.02	0	0.02	NA	NA
	Ethnicity “Asian”	0.516	0.02	0	0.02	NA	NA
	MELD score	0.569	0.01	0	0.01	NA	NA
	Underlying liver disease “alcohol induced liver disease”	0.507	0.01	0.01	0	NA	NA
	Ethnicity “Black”	0.515	0.01	0.01	0	NA	NA
	BCLC stage B	0.575	0.01	0	0.01	NA	NA
	BCLC stage C	0.523	0.01	0	0.01	NA	NA

A selection frequency of 1 indicates that this feature was consistently chosen in every instance for stratifying the lesion into either the complete response (CR) group or the non-complete response (non-CR) group

*ICC* intra-class correlation coefficient, *CI* confidence interval

## Discussion

In our study we developed different models to predict response of target lesions in patients with HCC who underwent radiation segmentectomy using baseline demographic and clinical data as well as radiomics from baseline MR images. The model using a combination of demographic/clinical data and radiomics from the PVP (Model D) showed the best performance with fair prediction power for CR at 6 months which was similar to the performance of using radiomics alone (Model C). On the other hand, baseline demographics/clinical parameters alone (Model A) and AFP alone (Model B) showed significantly worse performance compared to the models using radiomics (Models C, D, and E).

Quantitative studies evaluating HCC response prediction to radiation segmentectomy from MRI baseline images are sparse. To the best of our knowledge there are, to date, only a few studies that evaluated pre-radiation segmentectomy imaging findings in HCC patients using CT [22], FDG-PET/CT [37], SPECT CT [38] or MRI [21] to predict response and outcome, respectively. The study by Reiner et al. [22] found a sensitivity of 88% and a specificity of 75% for distinguishing responders from non-responders after radiation segmentectomy using histogram features quantification from baseline perfusion CT images [22] which was higher compared to our results (Model D, 70.6% and 66.2% respectively). In contrast to our study however, their study defined CR or PR as responders, whereas in our study only CR was considered as response. Notably, perfusion CT is not routinely used for radiation segmentectomy treatment planning in clinical practice. Jreige et al. [37] suggested that quantitative functional parameters from pre-radiation segmentectomy FDG-PET/CT studies such as SUVmax and tumor-to-liver uptake ratio are able to predict overall survival and progression-free survival. However, currently FDG-PET/CT plays a minor role in HCC evaluation and staging which might limit the role of functional parameters in clinical practice.

The investigation conducted by Marinelli et al. [21] yielded promising outcomes through the utilization of MRI radiomics features for prognosticating CR subsequent to radiation segmentectomy among patients with HCC, showcasing an AUC of 0.89, with a sensitivity of 80% and specificity of 88%. Notably, this performance surpassed that of baseline clinical characteristics (AUC 0.59, sensitivity 100%, specificity 28%). Nevertheless, in contrast to the methodology employed in our investigation, Marinelli et al. incorporated radiomics features extracted from both pre- and post-treatment MRI scans, a distinction that may account for the superior diagnostic efficacy observed in their study compared to ours. For our study, we opted to include only pre-TARE MRI data because we believe this approach is more practical in clinical practice compared to incorporating post-TARE images, as it would avoid subjecting the patient to an ineffective treatment and its associated potential side effects, and it may orient them towards other treatment modalities.

Another study by Chapiro et al. [39] found that in patients with HCC, the total and enhancing tumor volume from baseline MRI is associated with overall survival. Similarly, in our study a high tumor volume (represented by the feature “SHAPE_Volume”) was associated with absence of CR at 6 months after radiation segmentectomy with the highest single AUC. Our results reinforce previous results that tumor size or volume is one the most important predictors for treatment success when using intra-arterial therapies [39, 40].

A study by Hu et al. [19] found that, besides the factors such as tumor size, tumor vascularity and portal vein invasion, the number of tumors is a significant independent predictor of tumor response after chemoembolization in patients with unresectable HCC. In addition, Child–Pugh class B and C, and a high AFP value indicated poor prognosis for overall patient survival in that study. Similarly, in our study a high Child–Pugh score was associated with absence of CR and frequently selected by the prediction model with the best performance (Model D, clinical/demographics features and radiomics). Furthermore, in our study, lesions in patients with BCLC stage A had more frequently CR while, in contrast, lesions in patients with BCLC stage B, C and D showed more frequently non-CR. This may stem from the fact that the BCLC stage incorporates the number of lesions, lesion size and Child–Pugh class, which are all risk factors for treatment failure. Also, similar to the study by Hu et al. [19] we found that lower AFP at baseline was associated with CR at 6 months.

In our study, models using radiomics features performed better compared to models using demographic and clinical data alone. One explanation might be that radiomics features were extracted directly from the tumor, providing biological data specific to the target lesion. In contrast, many demographic and clinical features (e.g. age, gender, underlying liver disease, etc.) reflect broader patient-level biological data but may not directly capture the biology of the lesion itself. HCC is a complex disease with known heterogeneity in histological and molecular features, genetic alterations and oncogenic pathways influencing the disease prognosis [41, 42]. Radiomics technique can quantify tumor phenotypic heterogeneity and other imaging features that are not detectable by the human eye such as microvascular invasion [43, 44].

Current guidelines recommend that patients with early HCC should be evaluated for curative treatment options such as resection, transplantation or ablation, while arterially directed therapies (e.g. TACE or TARE/radiation segmentectomy) are indicated in patients with intermediate stage HCC who are not eligible for curative therapy or as a bridge to other curative therapies [2–4]. However, initial attempts to use segmental radiation segmentectomy as curative treatment for early HCC have been made [45]. Regardless of the intended treatment approach (curative or not), no prognostic score or evaluation method for response prediction after radiation segmentectomy are currently available. Therefore, our proposed model, using a combination of clinical/demographic features and radiomics may help in identifying those patients who might profit from additional or adjuvant therapy, such as immunotherapy [46].

As of now, the evidence for combining radiation segmentectomy and sorafenib or immunotherapy in patients with HCC is low [47–49]. However, initial results in patients with HCC showed immune activation in the local tumor microenvironment after radiation segmentectomy, suggesting that a combination of radiation segmentectomy and immunotherapy could improve patient outcomes [50]. Clinical trials have recently evaluated the combination of radiation segmentectomy and immunotherapy in patients with advanced HCC. Initial data show promising results with disease control rate of 82% and no increase of the adverse event rate [51]. However, final results of this clinical trial are not published yet.

Our study has several limitations. First, the retrospective design entails a potential selection bias. However, we made every effort in selecting consecutive patients treated with a similar technique (radiation segementectomy). Second, we did not validate our model in a separate validation set. This was mainly because the sample size of our cohort was not large enough to create an adequate training and validation sets. Therefore, we focused on constructing a robust model, using an elastic net with 100-fold cross validation for feature selection and model building. This could be peformed in a separate study. Third, in our study, radiation segmentectomy was very efficient with only a small number of lesions showing SD or even PD and these categories may therefore have been underrepresented. Fourth, we only assessed target lesion response at 6 months but did not assess long term response or survival. Fifth, we did not evaluate semantic features, as we wanted to develop methods that minimize reliance on subjective features.

In conclusion, a model consisting of radiomics features extracted from pre-radiation segmentectomy MRI and clinical parameters shows fair perforamance for predicting HCC response to radiation segmentectomy at 6 months. The performance of this model was significantly better compared to clinical parameters and serum AFP. These results need further independent validation.

## Supplementary Information

Below is the link to the electronic supplementary material.

Supplementary file1 (DOCX 28 KB)

## Abbreviations

ADC: Apparent diffusion coefficient
AFP: Alpha-fetoprotein
AUC: Area under curve
CR: Complete response
HBP: Hepatobiliary phase
HCC: Hepatocellular carcinoma
mRECIST: Modified response evaluation criteria in solid tumors
PD: Progressive disease
PR: Partial response
PVP: Portal venous phase
SD: Stable disease
TACE: Transarterial chemoembolization
TARE: Transarterial radioembolization
VOI: Volume of interest
^90^Y: Yttrium 90
