# Supplementary material for: Prediction of hepatocellular carcinoma response to radiation segmentectomy using an MRI-based machine learning approach
Source: Abdom Radiol (NY). 2024 Oct 26;50(5):2000–11. doi: 10.1007/s00261-024-04606-z (PMC11991973; doi:10.1007/s00261-024-04606-z)
Supplement: Supplementary file 1 — Supplementary file1 (DOCX 28 KB) [file 261_2024_4606_MOESM1_ESM.docx]

**Supplementary material**:

MRI parameters

The patients were scanned on either 1.5T MR systems (Magnetom Aera, Siemens Healthineers, n=45; Signa HDxt, GE Healthcare, n=20; Magnetom Avanto, Siemens Healthineers, n=20; Magnetom Amira, Siemens Healthineers, n=8; Optima MR450, GE Healthcare, n=8; Magnetom Symphony, Siemens Healthineers, n=2; Magnetom Espree, Siemens Healthineers, n=2; Ingenia, Philips, n=2; Signa Excite, GE Healthcare, n=1) or 3T MR systems (Magnetom Skyra, Siemens Healthineers, n=21; Discovery MR750, GE Healthcare, n=14; Magnetom Verio, Siemens Healthineers, n=7; Biograph, Siemens Healthineers, n=4). The most important MR parameter for the fat saturated T1-weighted sequence used for radiomics extraction are as follow:

For 1.5T systems: repetition time, range 3.07-5.07 ms; echo time, 1.25-2.39 ms; flip angel, 10-40°; slice thickness, 2.5-5.0 mm; number of averages, 0.7-1.

For 3T systems: repetition time, range 2.79-6.32 ms; echo time, 1.12-3.13 ms; flip angel, 9-12°; slice thickness, 2.5-4.0 mm; number of averages, 0.7-1.

mRECIST (modified Response Evaluation Criteria In Solid Tumors): Complete response (CR) was defined as disappearance of any intratumoral arterial enhancement in the target lesion; partial response (PR) as ≥30% diameter decrease of viable (enhancement in the arterial phase) target lesion (reference = target lesion baseline diameter); stable disease (SD) as any case not qualifying for PR or progressive disease (PD); and PD as ≥20% diameter increase of viable (enhancing in the arterial phase) target lesion (reference = smallest diameter of viable target lesion recorded since treatment start).

Image pre-processing: Before VOI placement and feature extraction, image pre-processing was performed as follows: 1) spatial resampling was set to 1 x 1 x 1 mm, 2) the number of grey-levels was set to n=64 for intensity discretization, and 3) for intensity rescaling the VOI values were automatically rescaled between mean -3∗standard deviations (SD) and mean +3∗SD of the voxel included in the VOI (voxels with an initial value < mean -3∗SD are set to mean -3∗SD; voxels with an initial value > mean +3∗SD are set to mean +3∗SD).

**Supplementary Table 1**: Inter- and intra-observer agreement for all radiomics features using the intraclass correlation coefficient (ICC) with 95% confidence intervals (95% CI).

|  | **Radiomics feature** | **Inter-reader** | | **Intra-reader** | |
| --- | --- | --- | --- | --- | --- |
|  |  | **ICC** | **95% CI** | **ICC** | **95% CI** |
| **Conventional indices** | CONVENTIONAL_#min | 0.997 | 0.995-0.998 | 0.963 | 0.934-0.079 |
|  | CONVENTIONAL_#mean | 1 | 1-1 | 1 | 1-1 |
|  | CONVENTIONAL_#std | 0.987 | 0.978-0.992 | 0.977 | 0.959-0.987 |
|  | CONVENTIONAL_#max | 1 | 1-1 | 1 | 0.999-1 |
| **First-order features (Histogram features)** | HISTO_Skewness | 0.938 | 0.899-0.962 | 0.950 | 0.912-0.972 |
|  | HISTO_Kurtosis | 0.874 | 0.794-0.922 | 0.947 | 0.907-0.970 |
|  | HISTO_ExcessKurtosis | 0.874 | 0.794-0.922 | 0.947 | 0.907-0.970 |
|  | HISTO_Entropy_log10 | 0.946 | 0.913-0.967 | 0.951 | 0.913-0.972 |
|  | HISTO_Entropy_log2 | 0.946 | 0.913-0.967 | 0.951 | 0.913-0.972 |
|  | HISTO_Energy | 0.943 | 0.909-0.965 | 0.928 | 0.872-0.959 |
| **Shape features** | SHAPE_Volume | 1 | 1-1 | 1 | 0.999-1 |
|  | SHAPE_Sphericity | 0.888 | 0.818-0.931 | 0.954 | 0.919-0.974 |
|  | SHAPE_Compacity | 0.999 | 0.999-1 | 0.999 | 0.999-1 |
| **Second-order features** | GLCM_Homogeneity | 0.949 | 0.917-0.968 | 0.970 | 0.947-0.983 |
|  | GLCM_Energy | 0.923 | 0.875-0.953 | 0.897 | 0.818-0.941 |
|  | GLCM_Contrast | 0.912 | 0.857-0.946 | 0.954 | 0.919-0.974 |
|  | GLCM_Correlation | 0.951 | 0.920-0.970 | 0.966 | 0.940-0.981 |
|  | GLCM_Entropy_log10 | 0.954 | 0.925-0.972 | 0.963 | 0.935-0.979 |
|  | GLCM_Entropy_log2 | 0.954 | 0.925-0.972 | 0.963 | 0.935-0.979 |
|  | GLCM_Dissimilarity | 0.935 | 0.894-0.960 | 0.959 | 0.928-0.977 |
|  | GLRLM_SRE | 0.962 | 0.938-0.977 | 0.979 | 0.964-0.988 |
|  | GLRLM_LRE | 0.964 | 0.942-0.978 | 0.974 | 0.955-0.985 |
|  | GLRLM_LGRE | 0.878 | 0.802-925 | 0.924 | 0.867-0.957 |
|  | GLRLM_HGRE | 0.898 | 0.834-0.937 | 0.961 | 0.932-0.978 |
|  | GLRLM_SRLGE | 0.878 | 0.802-925 | 0.920 | 0.859-0.955 |
|  | GLRLM_SRHGE | 0.934 | 0.893-0.960 | 0.966 | 0.941-0.981 |
|  | GLRLM_LRLGE | 0.884 | 0.811-0.929 | 0.936 | 0.888-0.964 |
|  | GLRLM_LRHGE | 0.967 | 0.946-0-980 | 0.978 | 0.961-0.987 |
|  | GLRLM_GLNU | 1 | 1-1 | 1 | 0.999-1 |
|  | GLRLM_RLNU | 1 | 1-1 | 1 | 0.999-1 |
|  | GLRLM_RP | 0.963 | 0.939-0.977 | 0.977 | 0.959-0.987 |
|  | NGLDM_Coarseness | 0.959 | 0.934-0.975 | 0.952 | 0.915-0.973 |
|  | NGLDM_Contrast | 0.811 | 0.693-0.884 | 0.835 | 0.709-0.906 |
|  | NGLDM_Busyness | 1 | 1-1 | 0.999 | 0.999-1 |
|  | GLZLM_SZE | 0.936 | 0.897-0.961 | 0.926 | 0.869-0.958 |
|  | GLZLM_LZE | 0.999 | 0.999-0.999 | 0.998 | 0.996-0.999 |
|  | GLZLM_LGZE | 0.558 | 0.280-0.728 | 0.813 | 0.670-0.894 |
|  | GLZLM_HGZE | 0.955 | 0.927-0.973 | 0.973 | 0.953-0.985 |
|  | GLZLM_SZLGE | 0.441 | 0.091-0.656 | 0.692 | 0.457-0.825 |
|  | GLZLM_SZHGE | 0.948 | 0.916-0.968 | 0.952 | 0.916-0.973 |
|  | GLZLM_LZLGE | 0.990 | 0.983-0.994 | 0.989 | 0.980-0.994 |
|  | GLZLM_LZHGE | 0.999 | 0.999-1 | 0.998 | 0.997-0.999 |
|  | GLZLM_GLNU | 1 | 1-1 | 1 | 0.999-1 |
|  | GLZLM_ZLNU | 0.999 | 0.999-1 | 1 | 0.999-1 |
|  | GLZLM_ZP | 0.951 | 0.920-0.970 | 0.974 | 0.954-0.985 |

**Supplementary Table 2:** Results from univariate analysis for clinical/demographic features and radiomics features with corresponding correlation with treatment response (Spearman correlation, r).

|  | **Feature** | **AUC** | **r** | **p-value*** |
| --- | --- | --- | --- | --- |
| **Clinical and demographic features** | Age | 0.503 | 0.005 | 0.944 |
|  | Gender | 0.542 | -0.089 | 0.249 |
|  | History of prior HCC | 0.507 | 0.021 | 0.783 |
|  | AFP | 0.619 | -0.193 | 0.012 |
|  | MELD score | 0.569 | -0.114 | 0.143 |
|  | Child-Pugh score | 0.591 | -0.159 | 0.039 |
|  | Number of target lesions | 0.533 | 0.082 | 0.291 |
|  | ^90^Y dose | 0.531 | 0.051 | 0.514 |
|  | No underlying liver disease | 0.505 | -0.027 | 0.727 |
|  | Underlying liver disease “HBV” | 0.516 | 0.040 | 0.609 |
|  | Underlying liver disease “HCV” | 0.517 | 0.031 | 0.687 |
|  | Underlying liver disease “cryptogenic cirrhosis” | 0.519 | -0.082 | 0.289 |
|  | Underlying liver disease “NASH” | 0.534 | -0.087 | 0.261 |
|  | Underlying liver disease “AIH” | 0.500 | -0.002 | 0.982 |
|  | Underlying liver disease “ASH” | 0.507 | 0.020 | 0.801 |
|  | Underlying liver disease “Other” | 0.509 | 0.077 | 0.324 |
|  | Ethnicity “White” | 0.531 | 0.059 | 0.445 |
|  | Ethnicity “Black” | 0.515 | -0.041 | 0.594 |
|  | Ethnicity “Asian” | 0.516 | -0.040 | 0.606 |
|  | Ethnicity “Hispanic” | 0.533 | 0.077 | 0.323 |
|  | Ethnicity “other” | 0.533 | -0.102 | 0.187 |
|  | BCLC stage 0 | 0.508 | 0.031 | 0.692 |
|  | BCLC stage A | 0.599 | 0.197 | 0.011 |
|  | BCLC stage B | 0.575 | -0.173 | 0.025 |
|  | BCLC stage C | 0.523 | -0.093 | 0.233 |
|  | BCLC stage D | 0.509 | -0.111 | 0.152 |
| **Radiomics features** | CONVENTIONAL_min | 0.528 | 0.046 | 0.557 |
|  | CONVENTIONAL_mean | 0.500 | -0.001 | 0.993 |
|  | CONVENTIONAL_std | 0.518 | -0.029 | 0.710 |
|  | CONVENTIONAL_max | 0.522 | -0.036 | 0.641 |
|  | HISTO_Skewness | 0.639 | -0.226 | 0.003 |
|  | HISTO_Kurtosis | 0.591 | 0.147 | 0.057 |
|  | HISTO_ExcessKurtosis | 0.591 | 0.147 | 0.057 |
|  | HISTO_Entropy_log10 | 0.655 | -0.252 | 0.001 |
|  | HISTO_Entropy_log2 | 0.655 | -0.252 | 0.001 |
|  | HISTO_Energy | 0.625 | 0.203 | 0.008 |
|  | SHAPE_Volume | 0.745 | -0.398 | <0.001 |
|  | SHAPE_Sphericity | 0.502 | -0.004 | 0.961 |
|  | SHAPE_Compacity | 0.740 | -0.390 | <0.001 |
|  | GLCM_Homogeneity | 0.618 | -0.192 | 0.013 |
|  | GLCM_Energy | 0.586 | 0.140 | 0.071 |
|  | GLCM_Contrast | 0.687 | 0.303 | <0.001 |
|  | GLCM_Correlation | 0.694 | -0.315 | <0.001 |
|  | GLCM_Entropy_log10 | 0.619 | -0.193 | 0.012 |
|  | GLCM_Entropy_log2 | 0.619 | -0.193 | 0.012 |
|  | GLCM_Dissimilarity | 0.673 | 0.281 | <0.001 |
|  | GLRLM_SRE | 0.631 | 0.213 | 0.006 |
|  | GLRLM_LRE | 0.630 | -0.212 | 0.006 |
|  | GLRLM_LGRE | 0.595 | 0.155 | 0.045 |
|  | GLRLM_HGRE | 0.551 | 0.083 | 0.288 |
|  | GLRLM_SRLGE | 0.599 | 0.160 | 0.038 |
|  | GLRLM_SRHGE | 0.650 | 0.243 | 0.001 |
|  | GLRLM_LRLGE | 0.510 | 0.016 | 0.841 |
|  | GLRLM_LRHGE | 0.640 | -0.228 | 0.003 |
|  | GLRLM_GLNU | 0.744 | -0.396 | <0.001 |
|  | GLRLM_RLNU | 0.743 | -0.395 | <0.001 |
|  | GLRLM_RP | 0.628 | 0.209 | 0.007 |
|  | NGLDM_Coarseness | 0.739 | 0.388 | <0.001 |
|  | NGLDM_Contrast | 0.720 | 0.358 | <0.001 |
|  | NGLDM_Busyness | 0.715 | -0.350 | <0.001 |
|  | GLZLM_SZE | 0.621 | 0.197 | 0.011 |
|  | GLZLM_LZE | 0.691 | -0.311 | <0.001 |
|  | GLZLM_LGZE | 0.582 | 0.134 | 0.084 |
|  | GLZLM_HGZE | 0.502 | 0.003 | 0.966 |
|  | GLZLM_SZLGE | 0.537 | 0.060 | 0.440 |
|  | GLZLM_SZHGE | 0.551 | 0.082 | 0.289 |
|  | GLZLM_LZLGE | 0.591 | -0.148 | 0.055 |
|  | GLZLM_LZHGE | 0.697 | -0.319 | <0.001 |
|  | GLZLM_GLNU | 0.732 | -0.377 | <0.001 |
|  | GLZLM_ZLNU | 0.731 | -0.376 | <0.001 |
|  | GLZLM_ZP | 0.659 | 0.259 | 0.001 |

*from Spearman correlation.
